# Clinical efficacy and safety analysis of aumolertinib in real-world treatment of EGFR-mutated advanced non-small-cell lung cancer

**DOI:** 10.3389/fphar.2024.1331138

**Published:** 2024-04-09

**Authors:** Xiaojuan Zhang, Mina Zhang, Xinyang Du, Guowei Zhang, Yuanyuan Niu, Chunhua Wei, Lanwei Guo, Chao Shi, Hangfan Liu, Huijuan Wang

**Affiliations:** ^1^ The Affiliated Cancer Hospital of Zhengzhou University & Henan Cancer Hospital, Shanghai, China; ^2^ Medical Oncology Scientific Group of the Central Medical Department, Jiangsu Hansoh, Pharmaceutical Group Co., Ltd., Shanghai, China

**Keywords:** non-small-cell lung cancer, EGFR, aumolertinib, elevated creatine kinase, T790M, brain metastasis

## Abstract

**Background:** This study aims to determine the efficacy and safety profile of aumolertinib in the real-word treatment setting for advanced non-small-cell lung cancer (NSCLC) patients harboring epidermal growth factor receptor (EGFR) mutations.

**Methods:** We retrospectively analyzed the clinical data of 173 EGFR-mutated advanced NSCLC patients who received aumolertinib treatment at Henan Cancer Hospital from April 2020 to December 2022. Progression-free survival (PFS) and overall survival (OS) were evaluated using Kaplan–Meier survival curves, while a Cox regression model was used for multifactorial analysis and prognostic factor assessment.

**Results:** Among patients administered first-line aumolertinib (n = 77), the objective remission rate (ORR) of 77.92% was observed, along with a disease control rate (DCR) of 100%. The median progression-free survival (mPFS) was 24.97 months, which did not reach the median overall survival (mOS). The patients treated with aumolertinib after progression on prior EGFR-tyrosine kinase inhibitor (TKI) therapy (n = 96) exhibited an ORR of 46.88%, a DCR of 89.58%, an mPFS of 15.17 months, and an mOS of 21.27 months. First-line treatment multivariate Cox regression analysis demonstrated a statistically significant impact of elevated creatine kinase on PFS (*p* = 0.016) and a similar significant influence of co-mutation on OS (*p* = 0.034). Furthermore, subsequent-line treatment multivariate Cox regression analysis showed a statistically significant impact of elevated creatine kinase on median PFS (*p* = 0.026) and a significant effect on the number of metastatic organs (*p* = 0.017), co-mutation (*p* = 0.035), and elevated creatine kinase (*p* = 0.014) on median OS.

**Conclusion:** Aumolertinib has shown clinical significance and can safely be used in the real-world setting for patients with EGFR mutation-positive NSCLC.

## Introduction

The advancement of genetic testing technology has paved the way for precise, and individualized targeted therapy to become a standard treatment approach for patients with advanced non-small-cell lung cancer (NSCLC) (Li et al., 2013). Consequently, the therapeutic strategy for advanced NSCLC patients harboring EGFR-positive mutations has transitioned from chemotherapy to tyrosine kinase inhibitors (TKIs), leading to a notable enhancement in patient survival time and quality of life (Mitsudomi et al., 2010; Rosell et al., 2012). Nonetheless, due to the drug resistance mechanisms and associated adverse events, most NSCLC patients treated with first- and second-generation EGFR-TKIs struggle to achieve progression-free survival (PFS) beyond 12 months, and this situation further exacerbate in patients with brain metastasis (Mok et al., 2009; Park et al., 2016; Westover et al., 2018; Zhao et al., 2019). The third-generation EGFR-TKI, osimertinib, was approved for the treatment of advanced NSCLC with EGFR-sensitive mutations and acquired T790M mutations, as well as for the postoperative adjuvant treatment of NSCLC driven by positive genes (Mok T. S. et al., 2017; Ramalingam et al., 2020; Tsuboi et al., 2023).

Aumolertinib, a potent and irreversible third-generation EGFR-TKI developed independently in China, can selectively inhibit EGFR-sensitive and T790M drug-resistant mutations (Lu et al., 2022a; Lu et al., 2022b). In the registered multi-center phase-III AENEAS clinical trials, aumolertinib extended the median progression-free survival (mPFS) by 9.4 months compared to gefitinib in NSCLC patients. The clinical trials of the aumolertinib study indicated that the most frequently reported adverse event was asymptomatic creatine kinase elevation (Yang et al., 2020; Lu et al., 2022a; Lu et al., 2022b). However, the efficacy and safety of aumolertinib in the real-world settings remain unknown, and whether superior to registered clinical trials and frequently reported adverse events are not clear. Prior research has established a correlation between creatine kinase elevation and EGFR-TKI benefits (Jiang et al., 2021). Moreover, other prognostic factors impacting EGFR-TKI, such as the number of metastatic organs and co-mutated genes, have also been documented (Oh et al., 2009; Park et al., 2013; Barnet et al., 2017). In this study, we performed a retrospective analysis to examine the efficacy and safety of aumolertinib in first-line and subsequent-line treatment in real-world settings for patients with advanced NSCLC, as well as identifying the potential clinical factors influencing the benefits of PFS and overall survival (OS) in aumolertinib-treated patients.

## Materials and methods

### Patient

In the current study we retrospectively analyzed patients with EGFR mutation-positive NSCLC who were treated with aumolertinib between April 2020 and December 2022 at the Affiliated Cancer Hospital of Zhengzhou University. The inclusion criteria were as follows: 1) patients must have been histologically or cytologically confirmed to have NSCLC at IIIB-IV stages (the 8th edition of tumor node metastasis classification), and stage ⅢB patients were those with non-surgically resectable tumors; 2) patients must have been confirmed to have EGFR mutation-positive NSCLC using next-generation sequencing (NGS) or polymerase chain reaction (PCR) techniques by histological or cytological samples from primary or metastatic lesions; 3) patients who had received aumolertinib therapy either alone or in combination with other treatments; 4) at least one measurable lesion as per the response evaluation criteria in solid tumors (RECIST 1.1); 5) the ages of the patients should be between 18 and 85 years, and Eastern Cooperative Oncology Group (ECOG) performance status was 0–3; and 6) patients must have completed course of treatment and follow-up data. This study was approved by the Ethics Committee of the Affiliated Cancer Hospital of Zhengzhou University (ethics no. 2020-329-002), and all patients or their respective families provided informed consent.

### Treatment and assessments

All patients were administered aumolertinib orally at a daily dosage of 110 mg. Among the participants, 44.51% of patients underwent first-line regimen treatment and had not previously been treated with EGFR-TKI, while the remaining 55.49% patients underwent prior EGFR-TKI therapy (including third-generation EGFR-TKI) and had shown resistance to these EGFR-TKIs. Follow-up assessments comprised a review of the outpatient and inpatient hospital information system (HIS) and telephone follow-up, with a cut-off date set as 28 February 2023. The median follow-up duration was 20.0 months. The imaging data were collected at baseline and 3 months after treatment and were used for initial evaluation. Subsequent efficacy evaluation was based on analyzing clinical images and follow-up in accordance with the RECIST1.1 criteria.

### Statistical analysis

Data were processed using SPSS 26.0 and GraphPad Prism 8 statistical software. Survival curves were plotted using the Kaplan–Meier method, with univariate analysis performed using the log-rank test. The Cox regression model was implemented for multifactorial analysis and to calculate the hazard ratio (HR) and the corresponding 95% confidence intervals, with a *p*-value less than 0.05 considered statistically significant.

## Results

### Baseline characteristics of patients

A total of 173 EGFR-mutant patients who had been treated with aumolertinib from April 2020 to December 2022 were included in the study. The median age was 60 years, and 93 patients (53.8%) were older than 60 years. In the total population, 98 patients (56.7%) had brain metastasis, and 166 (95.9%) cases were diagnosed with adenocarcinomas. Additionally, 94.8% of patients harbored exon 19 deletion (60.7%) or 21L858R mutations (34.1%), while 5.2% had rare mutations. Regarding the treatment regimen, 77 (43.8%) patients underwent first-line treatment, and the remaining 56.2% underwent other treatments. Notably, T790M-positive advanced NSCLC patients accounted for 33.3% of these cases. Concerning metastatic sites, 15.6% of patients harbored three or more metastatic organs. Among patients exhibiting compound mutations with other genes, the most common mutation was found in TP53. The detailed clinical characteristics of the patients are summarized in Table 1.

**TABLE 1:** Characteristics of all patients.

Characteristic n (%)			
Age (years)		Gene detection method	
Median (range)	60 (33–85)	PCR	55 (31.8%)
<60	80 (46.2%)	NGS	108 (62.4%)
≥60	93 (53.8%)	PCR/NGS	10 (5.8%)
Gender		Smoking status	
Male	55 (31.8%)	Yes	36 (20.8%)
Female	118 (68.2%)	No	137 (79.2%)
ECOG PS		T790M mutation	
0–1	138 (79.8%)	Yes	32 (33.3%)
≥2	35 (20.2%)	No/Unknown	64 (66.7%)
Brain and meningeal metastasis		Surgery	
Yes	98 (56.7%)	Yes	32 (18.5%)
No	75 (43.3%)	No	14 (81.5%)
Pathological type		Number of metastatic sites	
Adenocarcinoma	166 (95.9%)	0	6 (3.5%)
Squamous	2 (1.2%)	1	85 (49.1%)
Adenosquamous	2 (1.2%)	2	55 (31.8%)
Not-otherwise specified	3 (1.7%)	≥3	27 (15.6%)
EGFR mutation type		Line of aumolertinib treatment	
19Del	105 (60.7%)	1	77 (44.5%)
21L858R	59 (34.1%)	2	64 (37.0%)
Others	9 (5.2%)	3	19 (11.0%)
		≥4	13 (7.5%)
First-line treatment		Combined mutation	
Aumolertinib	77 (44.5%)	TP53	40 (23.1%)
Gefitinib	50 (28.9%)	CTNNB1	3 (1.7%)
Icotinib	28 (16.2%)	RB1	3 (1.7%)
Afatinib	8 (4.6%)	ERBB2	4 (2.3%)
Osimertinib	3 (1.7%)	KRAS	2 (1.2%)
Erlotinib	3 (1.7%)	PIK3CA	3 (1.7%)
Dacomitinib	3 (1.7%)	PTEN	2 (1.2%)
AZD3759	1 (0.6%)	EGFR amplification	3 (1.7%)

### Overall clinical benefit of aumolertinib

**FIGURE 1:**
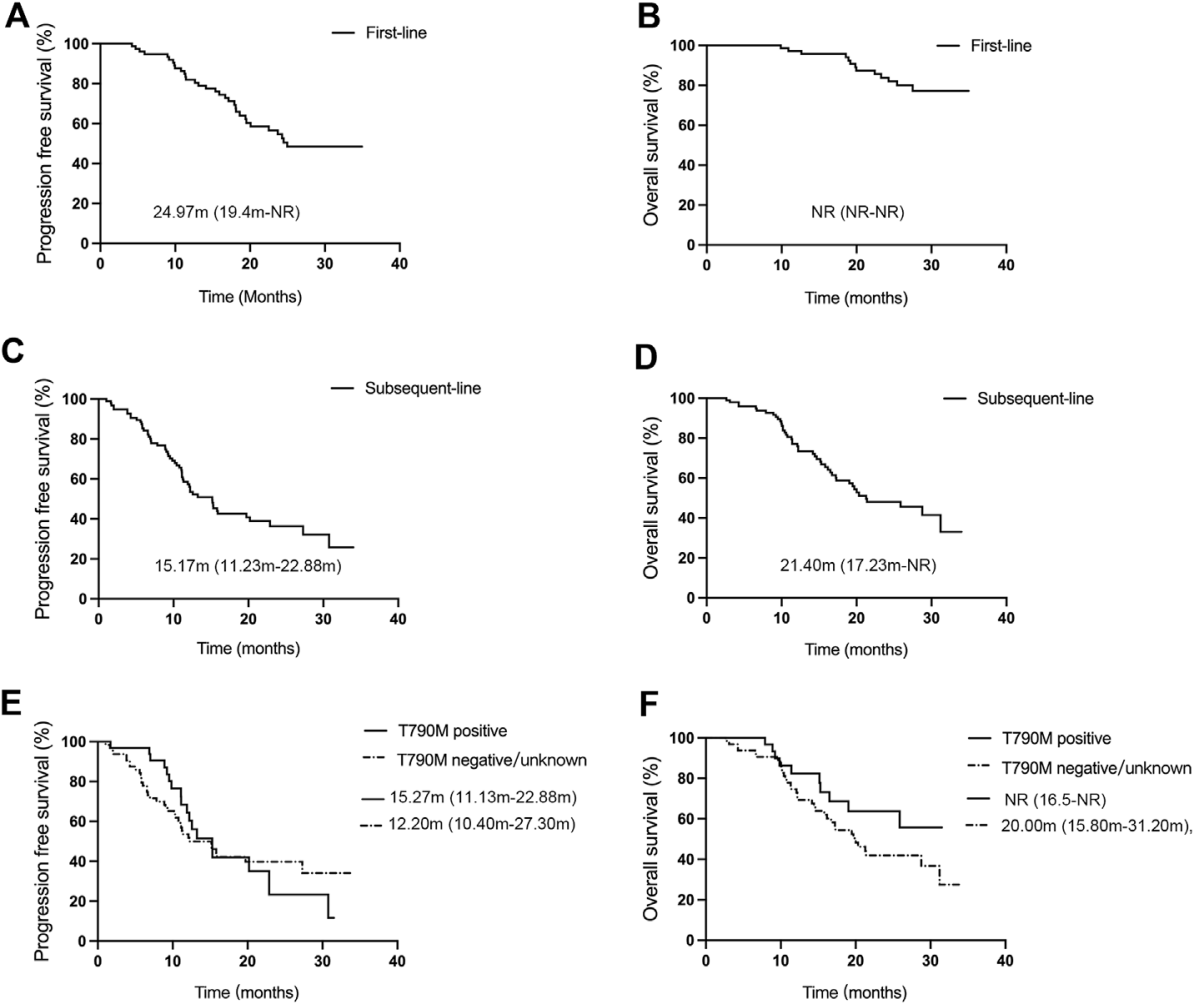
Efficacy evaluation of first-line and subsequent-line treatments with aumolertinib. **(A, B)** Kaplan–Meier analysis of progression-free survival and overall survival in patients treated with aumolertinib as the first-line therapy. **(C, D)** Kaplan–Meier analysis of progression-free survival and overall survival in patients treated with aumolertinib after EGFR-TKIs progressed. **(E, F)**. Kaplan–Meier analysis of progression-free survival and overall survival in T790M-positive and T790M negative/unknown patients.

The efficacy of treatment regimens was evaluated based on RESIST 1.1, which was divided into first-line and subsequent-line treatments with aumolertinib. As shown in Figure 1A, B, at the data cutoff of 28 February 2023, patients receiving first-line treatment (n = 77) with aumolertinib displayed an objective remission rate (ORR) and disease control rate (DCR) of 77.92% (95% CI: 67.02%–86.58%) and 100% (95% CI: 95.32%–100.00%), respectively; the mPFS was 24.97 months (95% CI: 19.4-not reached [NR]), and the mOS was NR (95% CI: NR-NR). Conversely, as presented in Figure 1C, D, patients treated with aumolertinib (n = 96) following progression on previous EGFR-TKI therapy showed an ORR of 46.88% (95% CI: 36.61%–57.33%), a DCR of 89.58% (95% CI: 81.67%–94.89%), an mPFS of 15.17 months (95% CI: 11.23–22.88), and a median overall survival (mOS) of 21.40 months (95% CI: 17.23-NR). The patients were divided into T790M-positive and T790M-negative groups according to the EGFR gene test. T790M-positive patients (n = 32) exhibited an ORR of 71.88% (95% CI: 53.25%–6.25%) and a DCR of 96.88% (95% CI: 83.78%–99.92%), and T790M-negative/unknown patients (n = 64) showed an ORR and DCR of 32.81% (95% CI: 21.59%–45.69%) and 85.94% (95% CI: 74.98%–93.36%), respectively.

The mPFS of T790M-positive and T790M-negative groups was 15.27 months (95% CI: 11.13–22.88) and 12.20 months (95% CI: 10.40–27.30), respectively; and the mOS was NR (95% CI: 16.5-NR) and 20.00 months (95% CI: 15.80–31.20) (Figure 1E, F). There was no significant difference found between the two groups concerning mPFS (*p* = 0.20) and mOS (*p* = 0.19).

### Clinical benefit of aumolertinib in patients with brain metastasis

**FIGURE 2:**
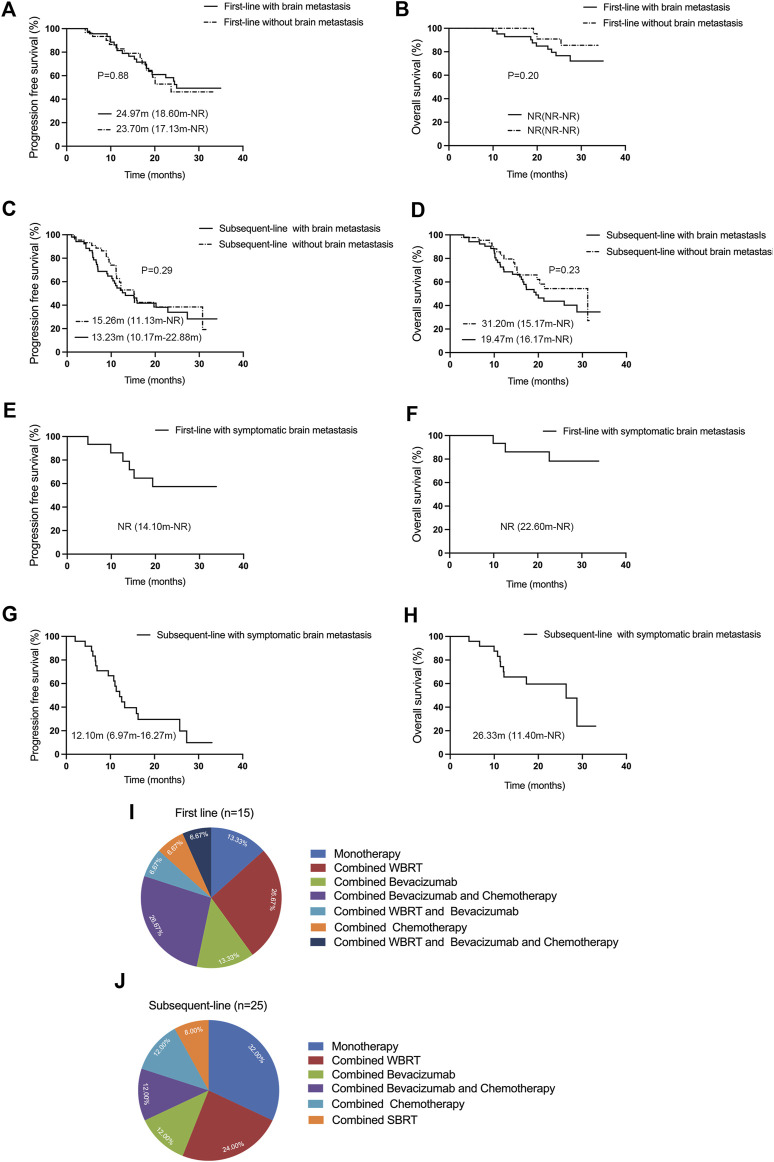
Efficacy evaluation of first-line and subsequent-line treatments with aumolertinib in patients with or without brain metastasis. **(A, B)** First-line therapy with aumolertinib Kaplan–Meier analysis of progression-free survival and overall survival in patients with or without brain metastasis. **(C, D)** Kaplan–Meier analysis of progression-free survival and overall survival in patients treated with aumolertinib after EGFR-TKIs progressed in patients with or without brain metastasis. **(E, F)** Kaplan–Meier analysis of progression-free survival and overall survival in patients with symptomatic brain metastasis undergoing first-line treatment with aumolertinib. **(G, H)** Kaplan–Meier analysis of progression-free survival and overall survival in patients with symptomatic brain metastasis undergoing subsequent-line treated with aumolertinib. **(I, J)** Detailed regimens of symptomatic brain metastasis patients in first-line and subsequent-line therapy.

Patients with EGFR mutations are more susceptible to developing brain metastasis, and the effectiveness of third-generation TKIs in patients with brain metastasis appears to surpass that of first- and second-generation TKIs. In our subset of patients given in Figure 2A, B, patients with brain metastasis (n = 46) who received first-line treatment exhibited an ORR of 84.78% (95% CI: 71.13%–93.66%), a DCR of 100% (95% CI: 92.29%–100%), an mPFS of 24.97 months (95% CI: 18.60-NR), but mOS was NR (95% CI: NR-NR). On the other hand, patients without brain metastasis (n = 31) demonstrated an ORR and DCR of 67.74% (95% CI: 48.63%–83.32%) and 100% (95% CI: 88.78%–100%), respectively; the mPFS was 23.70 months (95% CI: 17.13-NR), and mOS was NR (95% CI: NR-NR). Our results indicated that patients with brain metastasis experienced better clinical benefit from first-line aumolertinib treatment. However, these benefits did not statistically differ from those patients without brain metastasis in terms of PFS and OS (*p* = 0.88; *p* = 0.20). Among cases undergoing subsequent-line treatment given in Figure 2C, D, patients with brain metastasis (n = 52) had an ORR and DCR of 36.54% (95% CI: 23.62%–51.04%) and 86.54% (95% CI: 74.21%–94.41%), respectively, and the mPFS and mOS were 13.23 months (95% CI: 10.17–22.88) and 19.47 months (95% CI: 16.17-NR), respectively. In contrast, patients without brain metastasis (n = 44) presented an ORR of 56.82% (95% CI: 41.03%–71.65%), a DCR of 93.18% (95% CI: 81.34%–98.57%), an mPFS of 15.26 months (95% CI: 11.13-NR), and an mOS of 31.20 months (95% CI: 15.17-NR). Differences in PFS and OS between patients with and without brain metastasis in the subsequent-line treatment were not statistically significant (*p* = 0.29, *p* = 0.23). In general, aumolertinib exhibited encouraging clinical outcomes in patients with or without brain metastasis, particularly in those receiving first-line treatment.

In real-world settings, some patients with brain metastasis exhibit associated clinical symptoms such as dizziness, nausea, and vomiting. In this study, we conducted a pioneering analysis of aumolertinib efficacy in patients with clinically symptomatic brain metastasis. As demonstrated in Figure 2E, F, the patients with symptomatic brain metastasis (n = 15) who received first-line aumolertinib treatment achieved an ORR of 86.67% (95% CI: 59.54%–98.34%), a DCR of 100% (95% CI: 78.20%–100%), but the mPFS (95% CI: 14.10-NR) and mOS (95% CI: 22.60-NR) were NR. Similarly, as shown in Figure 2G, H, the ORR and DCR of patients with symptomatic brain metastasis (n = 25) who received subsequent-line treatment with aumolertinib were 48.0% (95% CI: 27.80%–68.70%) and 96.00% (95% CI: 79.65%–99.90%), respectively, and the mPFS and mOS were 12.10 months (95% CI: 6.97–16.27) and 26.33 months (95% CI: 11.40-NR), respectively. Significant symptomatic relief was observed as a result of both first-line and subsequent-line treatments. As illustrated in Figure 2I, the predominant treatment regimens for patients with symptomatic brain metastasis in the first-line treatment include monotherapy (n = 2), combined whole brain radiotherapy (WBRT, n = 4), combined bevacizumab (n = 2), combined bevacizumab and chemotherapy (n = 4), combined WBRT and bevacizumab (n = 1), combined chemotherapy (n = 1), and combined WBRT and bevacizumab and chemotherapy (n = 1). As shown in Figure 2J, for patients with symptomatic brain metastasis receiving subsequent-line treatment, the primary treatment regimens included monotherapy (n = 8), combined WBRT (n = 6), combined bevacizumab (n = 3), combined WBRT and bevacizumab (n = 3), combined chemotherapy (n = 3), and combined stereotactic body radiation therapy (SBRT, n = 2).

### Analysis of influencing factors for mPFS and mOS in the first-line aumolertinib treatment

We conducted univariate and multivariate Cox regression analyses to understand the factors potentially impacting the PFS and OS in aumolertinib treatment. Moreover, we executed multifactorial analyses on univariate analysis with a *p*-value of less than 0.1, considered candidate prognostic factors.

**FIGURE 3:**
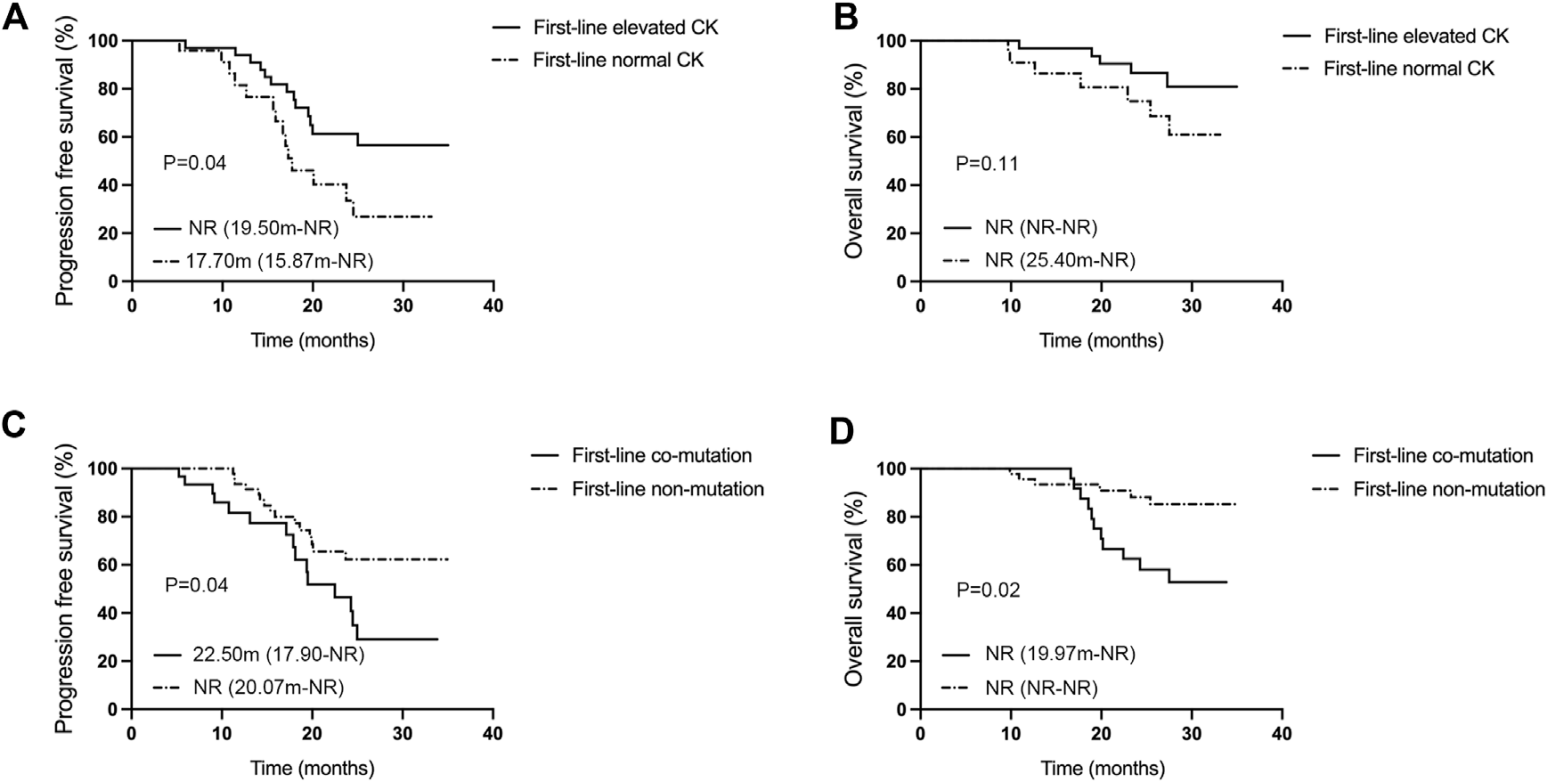
Analysis of influencing factors for mPFS and mOS in the first-line aumolertinib treatment. **(A, B)** Kaplan–Meier analysis of progression-free survival and overall survival in patients with or without creatine kinase elevation undergoing first-line treatment with aumolertinib. **(C, D)** Kaplan–Meier analysis of progression-free survival and overall survival in patients treated with aumolertinib after EGFR-TKIs progressed in patients with or without co-mutation.

The multivariate Cox regression analysis outcomes indicated a statistically significant effect of elevated creatine kinase on PFS (*p* = 0.032, Table 2). The effect of EGFR mutations (*p* = 0.019), the number of metastases (*p* = 0.019), and co-mutations (*p* = 0.034) on mOS was also statistically significant (Table 3). Efficacy assessment (Figure 3A, B) revealed that for patients undergoing first-line aumolertinib treatment, those with elevated creatine kinase (n = 33) showed an ORR of 78.79% (95% CI: 61.09%–91.02%), a DCR of 100% (95% CI: 89.42%–100%), and unreached mPFS (95% CI: 19.50-NR) and mOS (95% CI: NR-NR). The patients with non-elevated creatine kinase (n = 24) demonstrated an ORR and DCR of 83.33% (95% CI: 62.62%–95.27%) and 100% (95% CI: 85.75%–100%), respectively; the mPFS was 17.70 months (95% CI: 15.87-NR); but the mOS was NR (95% CI: 22.9-NR). A statistically significant difference in mPFS was observed between the two groups (*p* = 0.04), whereas the mOS was not statistically significant (*p* = 0.11). Patients with co-mutations (n = 30, Figure 3C, D) demonstrated an ORR of 83.33% 95% CI: (65.28%–94.36%), a DCR of 100% (95% CI: 88.43%–100%), an mPFS of 22.50 months (95% CI: 17.90-NR), and mOS was NR (95% CI: 19.97-NR), while patients without co-mutations (n = 47) exhibited an ORR of 74.47% (95% CI: 59.65%–86.06%), a DCR of 100% (95% CI: 92.45%–100%), and mPFS (95% CI: 20.07-NR) and mOS (95% CI: NR-NR) were NR. The difference in mPFS and mOS between the two groups was statistically significant (*p* = 0.04 and *p* = 0.02, respectively). Given that TP53 is the most common mutation in our study, we also analyzed the effect of TP53 on first-line treatment (Supplementary Figure S1A, B). The patients with TP53 (n = 19) showed an mPFS of 19.40 months (95% CI: 13.10–24.98), and mOS was NR (95% CI: 19.17-NR), while for patients without TP53 (n = 51), the mPFS (95% CI: 18.60-NR) and mOS (95% CI: NR-NR) were NR. There was no significance in mPFS and mOS between the two groups (*p* = 0.11 and *p* = 0.34, respectively). Thus, elevated creatine kinase serves as a crucial predictor of mPFS extension with first-line aumolertinib treatment, while co-mutation stands as a significant prognostic factor for mOS with first-line aumolertinib treatment.

**TABLE 2:** Univariate analysis and multivariate analysis for influencing factors of first-line treatment progression-free survival.

Factor	Univariate analysis			Multivariate analysis		
	HR	95% CI	P	HR	95% CI	P
Gender (male vs. female)	1.864	0.906–3.837	0.091	1.431	0.180–11.371	0.735
Age (≥60 years old vs. < 60 years old)	0.920	0.459–1.842	0.813			
Smoking (yes vs. no)	2.371	1.042–5.392	0.040	6.726	0.779–58.108	0.083
ECOGPS (2 vs. 0–1)	1.325	0.595–2.952	0.491			
EGFR mutation (21L858R vs. 19Del)	0.889	0.474–1.669	0.715			
Metastasis number (≥3 vs. < 3)	2.453	1.203–5.001	0.014	2.221	0.938–5.260	0.070
Brain metastasis (yes vs. no)	0.931	0.453–1.912	0.931			
Concomitant mutation (yes vs. no)	1.913	0.947–3.863	0.070	1.256	0.566–2.788	0.575
Creatine kinase elevation (yes vs. no)	2.592	1.192–5.636	0.016	2.462	1.081–5.606	0.032

**TABLE 3:** Univariate analysis and multivariate analysis for influencing factors of first-line treatment overall survival.

Factor	Univariate analysis			Multivariate analysis		
	HR	95% CI	P	HR	95% CI	P
Gender (male vs. female)	1.735	0.567–5.314	0.334			
Age (≥60 years old vs. < 60 years old)	1.420	0.464–4.343	0.538			
Smoking (yes vs. no)	1.616	0.444–5.889	0.467			
ECOGPS (2 vs. 0–1)	1.024	0.281–3.724	0.972			
EGFR mutation (21L858R vs. 19Del)	2.029	0.876–4.699	0.099	3.122	1.209–8.060	0.019
Metastasis number (≥3 vs. < 3)	2.836	0.952–8.446	0.061	3.897	1.255–12.106	0.019
Brain metastasis (yes vs. no)	2.156	0.593–7.837	0.243			
Concomitant mutation (yes vs. no)	2.951	0.989–8.807	0.049	3.312	1.092–10.043	0.034
Creatine kinase elevation (yes vs. no)	0.483	0.140–1.672	0.251			

### Analysis of influencing factors for mPFS and mOS in the subsequent-line aumolertinib treatment

The multivariate Cox regression analysis disclosed that elevated creatine kinase (*p* = 0.026) had a statistically significant impact on mPFS (Table 4). Performance status (PS) score (*p* = 0.011), the number of metastases (*p* = 0.017), co-mutations (*p* = 0.035), and elevated creatine kinase (*p* = 0.014) demonstrated a statistically significant influence on mOS (Table 5).

**TABLE 4:** Univariate analysis and multivariate analysis for influencing factors of subsequent-line treatment progression-free survival.

Factor	Univariate analysis			Multivariate analysis		
	HR	95% CI	P	HR	95% CI	P
Gender (male vs. female)	1.972	1.153–3.371	0.013	1.252	0.670–2.342	0.481
Age (≥60 years old vs. < 60 years old)	1.467	0.857–2.510	0.163			
Smoking (yes vs. no)	1.467	0.790–2.667	0.230			
ECOGPS (2 vs. 0–1)	1.698	0.949–3.040	0.075	1.923	0.873–4.234	0.105
Metastasis number (≥3 vs. < 3)	1.871	0.957–3.659	0.067	1.445	0.691–3.022	0.327
T790M mutation (yes vs. no/unknown)	0.928	0.527–1.633	0.795			
Concomitant mutation (yes vs. no)	1.963	1.143–3.372	0.015	1.664	0.904–3.063	0.102
Brain metastasis (yes vs. no)	1.415	0.816–2.456				
Creatine kinase elevation (yes vs. no)	0.481	0.251–0.922	0.027	0.449	0.222–0.908	0.026

**TABLE 5:** Univariate analysis and multivariate analysis for influencing factor of subsequent-line treatment overall survival.

Factor	Univariate analysis			Multivariate analysis		
	HR	95% CI	P	HR	95% CI	P
Gender (male vs. female)	1.840	1.010–3.351	0.046	1.397	0.482–3.946	0.549
Age (≥60 years old vs. < 60 years old)	1.185	0.655–2.146	0.574			
Smoking (yes vs. no)	2.304	1.209–4.391	0.011	1.573	0.162–2.033	0.389
ECOGPS (2 vs. 0–1)	1.820	0.967–3.422	0.063	3.330	1.313–8.442	0.011
Metastasis number (≥3 vs. < 3)	4.134	2.085–8.198	0.000	2.907	1.209–6.990	0.017
T790M mutation (yes vs. no/unknown)	0.605	0.299–1.222	0.161			
Concomitant mutation (yes vs. no)	2.088	1.158–3.767	0.014	2.119	1.056–4.580	0.035
Brain metastasis (yes vs. no)	1.884	0.988–3.593	0.045	1.203	0.521–2.781	0.665
Creatine kinase elevation (yes vs. no)	0.374	0.180–0.778	0.008	0.335	0.140–0.805	0.014

**FIGURE 4:**
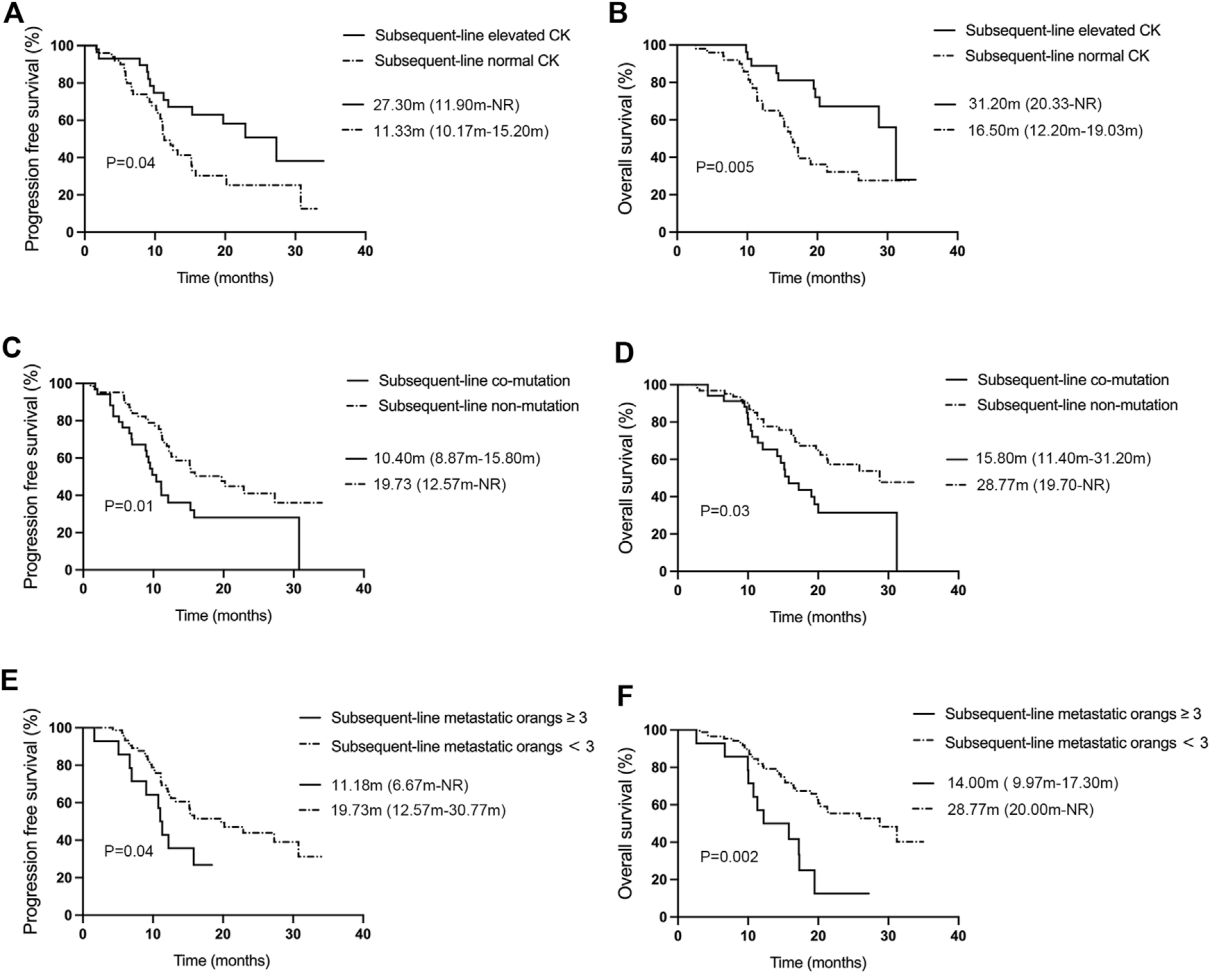
Analysis of influencing factors for mPFS and mOS of aumolertinib treatment in the subsequent-line therapy. **(A, B)** Kaplan–Meier analysis of progression-free survival and overall survival with or without creatine kinase elevation in patients undergoing first-line treatment with aumolertinib. **(C, D)** Kaplan–Meier analysis of progression-free survival and overall survival in patients treated with aumolertinib after EGFR-TKIs progressed in patients with or without co-mutation. **(E, F)** Kaplan–Meier analysis of progression-free survival and overall survival in patients treated with aumolertinib after EGFR-TKIs progressed in patients with metastatic organs more than three or not.

The efficacy analysis (Figure 4A, B) showed that subsequent-line treatment patients treated with aumolertinib who had elevated creatine kinase (n = 29) showed an ORR of 51.72% (95% CI: 32.53%–70.55%), a DCR of 93.10% (95% CI: 77.23%–99.15%), and mPFS and mOS were 27.30 months (95% CI: 11.90-NR) and 31.20 months (95% CI: 20.33-NR), respectively. In contrast, patients without elevated creatine kinase (n = 50) demonstrated an ORR of 48.00% (95% CI: 33.66%–62.59%), a DCR of 92.00% (95% CI: 80.77%–97.78%), and mPFS and mOS were 11.33 months (95% CI: 10.17–15.20) and 16.50 months (95% CI: 12.20–19.03), respectively. Both PFS and OS differences between these groups were statistically significant (*p* = 0.04, *p* = 0.005). Patients with co-mutations (n = 34, Figure 4C, D) showed an ORR of 41.18% (95% CI: 24.65%–59.30%), a DCR of 79.41% (95% CI: 24.65%–59.30%), and mPFS and mOS were 10.40 months (95% CI: 8.87–15.80) and 15.80 months (95% CI: 11.40–31.20), respectively; while those without co-mutations (n = 62) showed an ORR of 48.38% (95% CI: 35.50%–61.44%), a DCR of 95.16% (95% CI: 86.50%–98.99%), an mPFS of 19.73 months (95% CI: 12.57-NR), and an mOS of 28.77 months (95% CI: 19.70-NR). Both mPFS and mOS differences were statistically significant (*p* = 0.01, *p* = 0.03). We also analyzed the effect of TP53 on subsequent-line therapy (Supplementary Figure S1C, D). The patients with TP53 (n = 21) showed an mPFS of 10.40 months (95% CI: 5.90–19.50) and an mOS of 14.67 months (95% CI: 10.40–19.47), while patients without TP53 exhibited an mPFS of 15.90 months (95% CI: 12.20-NR) and an mOS of 28.77 months (95% CI: 20.33-NR). There was a statistical difference between the mPFS and mOS in the two groups (*p* = 0.01, *p* = 0.002). Figures 4E, F reveal that patients undergoing subsequent-line aumolertinib treatment with a number of metastatic organs ≥3 (n = 14) demonstrated an ORR of 21.42% (95% CI: 4.66%–50.80%), a DCR of 92.86% (95% CI: 66.13%–99.82%), an mPFS of 11.18 months (95% CI: 6.67-NR), and an mOS of 14.00 months (95% CI: 9.97–17.30). Patients with less than three metastatic organs (n = 82) showed an ORR of 50.00% (95% CI: 38.75%–61.25%), a DCR of 89.02% (95% CI: 80.19%–94.86%), an mPFS of 19.73 months (95% CI: 12.57–30.77), and an mOS of 28.77 months (95% CI: 20.00-NR). The differences in PFS and OS between these two groups were statistically significant (*p* = 0.04, *p* = 0.002).

### Adverse events

The major adverse events associated with oral aumolertinib therapy in this investigation are shown in Table 6. The adverse events included elevated creatine kinase (35.83%), rash (9.2%), pruritus (6.4%), increased AST (6.4%), increased ALT (6.9%), leukopenia (5.8%), anemia (3.5%), stomatitis (2.3%), and diarrhea (4.6%). The majority of these adverse events were categorized as grades 1–2, and grade 3 or higher adverse events were limited to an increase in creatine kinase (2.9%) and anemia (1.2%). These adverse events were symptomatically managed, leading to an amelioration of biochemical indices and clinical symptoms, without necessitating any dosage reduction or discontinuation due to adverse reactions.

**TABLE 6:** Treatment-related adverse events of all the patients.

Adverse event	n	Grade 1–2, n (%)	Grade 3–4, n (%)
Creatine kinase elevation	62	57 (32.9%)	5 (2.9%)
Erythra	16	16 (9.2%)	0
Skin pruritus	10	10 (5.8%)	0
AST elevation	11	11 (6.4%)	0
ALT elevation	12	12 (6.9%)	0
Leucopenia	10	10 (5.8%)	0
Anemia	6	4 (2.3%)	2 (1.2%)
Stomatitis	4	4 (2.3%)	0
Diarrhea	8	8 (4.6%)	0

## Discussion

This retrospective study assessed the efficacy of aumolertinib in first-line and subsequent-line patients in the real-world settings and on further discernment of the factors influencing PFS and OS. We found that the the mPFS of first-line and subsequent-line patients who were treated with aumolertinib was 24.97 months and 15.17 months, respectively. Influencing factors for PFS and OS were also analyzed using the COX model, and factors impacting the efficacy of aumolertinib were scrutinized including brain metastasis, elevated creatine kinase, and co-mutations. The outcomes revealed that elevated creatine kinase, the number of metastases, and co-mutations all exerted a measurable influence on the efficacy of aumolertinib. Our data demonstrate excellent efficacy and safety profiles in our study and surpassed that of registered clinical trials.

Since the introduction of EGFR-TKI, its efficacy on brain metastasis has attracted considerable attention. The third-generation EGFR-TKI displayed enhanced intracranial efficacy compared to its first- and second-generation counterparts, but some registered clinical trials excluded largely patients with brain metastasis (Wu et al., 2017). In the CNS complete analysis set (CFAS) of the AENEAE study, aumolertinib has shown good results in patients with brain metastasis and boasted an mPFS of 29.0 months. Importantly, we also scrutinized the efficacy of EGFR-TKI in patients with symptomatic brain metastasis for the first time to explore the benefits of EGFR-TKI in this patient population. The results demonstrated that either aumolertinib monotherapy or combination therapy provides substantial benefits in both first-line and subsequent-line treatment.

In our population with subsequent-line treatment, aumolertinib has demonstrated notable clinical activity against T790M-negative/unknown patients. Nonetheless, previously, patients with T790M-negative mutations were administered chemotherapy or combination with immunotherapy (Soria et al., 2015; Mok T. S. K. et al., 2017; Socinski et al., 2018). The previous EGFR-TKI studies were carried out to examine the efficacy of EGFR-TKIs in T790M-negative/unknown patients. In multi-center phase-II AURA1 and TREM studies, osimertinib achieved an ORR of 20% and an ORR of 28% in T790M-negative patients (Janne et al., 2015; Eide et al., 2020). A Phase-II investigation (WJOG12819L) that explored osimertinib for treating T790M-negative mutation reported an mPFS of 4.07 months and mOS of 13.73 months (Takeda et al., 2023). In this study, we observed a longer mPFS (12.20 months) and mOS (20.00 months). However, it is still not very clear how T790M-negative/unknown patients were benefited from aumolertinib. According to the detailed information on patients, only 16.7% of patients were analyzed by tissue biopsies, remaining were analyzed by blood samples. Given the false-negative rate of plasma T790M testing and tumor tissue heterogeneity, we believe that the efficacy is a bit exaggerated in previous T790M-negative/unknown patients (Sacher et al., 2016). Aumolertinib showed less toxicity and better efficacy than chemotherapy; it may provide a viable treatment option for T790M-negative patients, particularly those with brain metastasis or reluctant to undertake chemotherapy.

Elevated creatine kinase (CK) is the most frequent adverse event related to aumolertinib treatment. In this study, a total of 136 patients have detailed CK test results, and the majority of them showed high CK levels without any clinical symptoms, which is similar to previous studies (Lu et al., 2022a). The potential prognostic value of CK levels has been reported previously, and the AENEAS study has examined the correlation of elevated CK with extended PFS (Adenis et al., 2012; Jiang et al., 2021; Xing Ru-yue, 2022). In our study, elevated CK was identified as a significant predictor of prolonged median PFS in patients undergoing treatment with aumolertinib, both as a first-line and subsequent-line treatment. Although the pathophysiological mechanisms that underpin this correlation remain to be fully elucidated, it has the potential to facilitate further execution of personalized clinical treatment because of detection convenience.

Previous studies have indicated that co-mutation genes are closely related to histopathological manifestations, tumor microenvironment, acquired drug resistance mechanism, clinical benefit, and prognosis (Skoulidis and Heymach, 2019). The clinical research studies indicated that co-mutated genes can attenuate the efficacy of EGFR-TKI because of changing the molecular conformation of EGFR tyrosine kinase’s structural domains and clinical heterogeneity (VanderLaan et al., 2017; Harrison et al., 2020; Kitadai and Okuma, 2022). With the current treatment landscape, the common concurrent genetic alterations (TP53, PIK3CA, and PTEN) and concurrent driver gene alterations (ALK, KRAS, ROS1, and MET) should be concerned (Guo et al., 2020). Furthermore, specific gene’s co-variation could be better than that of several concomitant altered genes in predicting EGFR-TKI efficacy (Zhu et al., 2021). In our study, patients without co-mutations in the subsequent-line aumolertinib treatment exhibited approximately double mPFS and mOS in comparison to those patients with co-mutations. The most common co-mutation in our study was TP53 (23.1%), and we found that TP53 had an effect on the PFS and OS of subsequent-line but not first-line treatment. A more comprehensive understanding of the relationship between specific co-mutations and TKI efficacy would undoubtedly assist in predicting clinical outcomes and selecting the most optimal treatment strategy for patients presenting with these co-mutations. Studies have shown that metastasis in the number of organs may impact EGFR-TKI treatment efficacy and survival post-failure (Oh et al., 2009; Lin et al., 2016). In our study, patients with equal or more than three metastatic organs and less than three showed substantial differences in PFS and OS. However, these influencing factors were not evident in patients receiving first-line therapy, probably due to different resistance mechanisms or co-mutation with unknown genes. Hence, the first-line therapy of aumolertinib has great significance in the treatment of patients with EGFR-positive mutations.

Nevertheless, though this study provides meaningful data, we still acknowledge the several limitations. First, this study is a single-center, retrospective design, and has a relatively limited number of samples. Second, the timing of transition and discretion in therapy according to the different attending physicians and the patient’s preference and the selection of drugs administered with aumolertinib monotherapy or combination therapy were influenced. Additionally, some data of mOS were not ascertained due to the relatively short follow-up period, and more censored events may have influenced on the OS at subsequent follow-up. As a result, longer-term follow-ups and multicentered studies are warranted to further substantiate these findings.

## Conclusion

Aumolertinib demonstrated considerable efficacy and safety in both first-line and subsequent-line treatments of patients with EGFR-mutated NSCLC patients. Increased CK could be used as a clinical indicator, and the presence of co-mutations and a larger number of metastatic organs are critical factors influencing the unfavorable prognosis of aumolertinib treatment. Special attention should be paid to the treatment of patients with poor prognosis. Further prospective and multicenter clinical trials are warranted to accumulate evidence, demonstrating the effectiveness of aumolertinib for EGFR-mutated advanced NSCLC patients with co-mutations and multiple metastasis.

## Data Availability

The raw data supporting the conclusion of this article will be made available by the authors, without undue reservation.
