# Supplementary material for: Clinical efficacy and safety analysis of aumolertinib in real-world treatment of EGFR-mutated advanced non-small-cell lung cancer
Source: Front Pharmacol. 2024 Apr 9;15:1331138. doi: 10.3389/fphar.2024.1331138 (PMC11036126; doi:10.3389/fphar.2024.1331138)

SUPPLEMENTARY FIGURE S1. The efficacy evaluation of first-line and subsequent-line treatment with Aumolertinib with or without TP53 mutation patients. (A, B) The first-line therapy with Aumolertinib Kaplan-Meier analysis of progression free survival and overall survival in patients with or without TP53 mutation. (C, D) Kaplan-Meier analysis of progression free survival and overall survival in patients treated with Aumolertinib after EGFR-TKIs progressed in patients with or without or without TP53 mutation.

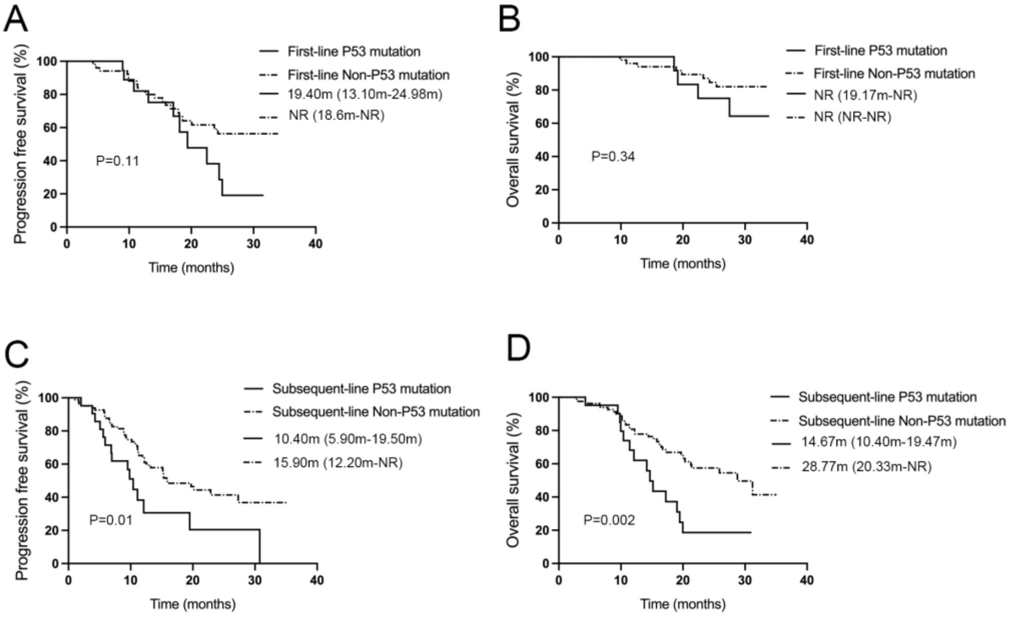

Supplement: Supplementary file 1 [file DataSheet1.pdf]
